# “It’s going to be hard you know…” Teachers’ perceived role in widening access to medicine

**DOI:** 10.1007/s10459-020-09984-9

**Published:** 2020-07-25

**Authors:** Kirsty Alexander, Sandra Nicholson, Jennifer Cleland

**Affiliations:** 1 Research Department of Medical Education (RDME), UCL Medical School, University College London (UCL), London, UK; 2 Centre for Medical Education, Institute of Health Sciences Education, Barts and the London School of Medicine and Dentistry, Queen Mary University of London, London, UK; 3 Medical Education Research and Scholarship Unit (MERSU), Lee Kong Chian School of Medicine, Nanyang Technological University Singapore, Singapore, Singapore

**Keywords:** Widening access, Widening participation, Medical school, Teachers, High school, Interviews, Diversity, Barriers

## Abstract

Medical schools worldwide undertake widening access (WA) initiatives (e.g. pipeline, outreach and academic enrichment programmes) to support pupils from high schools which do not traditionally send high numbers of applicants to medicine. UK literature indicates that pupils in these schools feel that their teachers are ill-equipped, cautious or even discouraging towards their aspiration and/or application to medicine. This study aimed to explore teachers’ perspectives and practices to include their voice in discussions and consider how medical schools might best engage with them to facilitate WA. Interviews were conducted with high school teachers in three UK regions, working in schools targeted by WA initiatives. Data were analysed thematically using template analysis, using a largely data-driven approach. Findings showed that although medicine was largely seen as a prestigious and worthwhile career, teachers held reservations about advocating this above other choices. Teachers saw it as their role to encourage pupils to educate themselves about medicine, but to ultimately allow pupils to make their own decisions. Their attitudes were influenced by material constraints in their schools, and the perception of daunting, long and emotionally difficult admissions requirements, with low chances of success. Medical schools may wish to work with teachers to understand their hesitations and help them develop the mindset required to advocate a challenging and unfamiliar career, emphasising that this encouragement can further the shared goal of empowering and preparing pupils to feel capable of choosing medicine. Reciprocally, medical schools should ensure pupils have fair opportunities for access, should they choose to apply.

## Introduction

Globally, the medical profession consists predominantly of individuals from affluent backgrounds, often educated in schools which outperform the average (AFMC 2010; Department of Education and Training 2019; Milburn 2012). School disparities are reflected in medical school applications. For example, 80% of UK medical school applicants come from only 20% of UK high schools, and half of schools have sent no applicants to medicine in recent years (Medical Schools Council 2014a, b); 44% of applicants come from grammar (academically selective) and independent (fee-paying) schools (Mathers et al. 2016), whilst only approximately 6.5% of the school age population attend these schools (Bolton 2017; ISC 2020).

The lack of socioeconomic diversity in medicine has caused concern that medical schools may not be promoting social mobility nor creating the best possible workforces to care for the populations they serve (Larkins et al. 2015; Medical Schools Council 2014a; Milburn 2012; O’Connell et al. 2017; Puddey et al. 2017). As applicants from independent and grammar schools are only slightly more likely to be accepted for a place at medical school in comparison to those applying from state (free to attend) schools (Steven et al. 2016), there have been strong calls for the applicant pool to become more representative of the population as a whole (Mathers et al. 2011; McLachlan 2005; O’Neill et al. 2013). This has caused an increase in medical schools’ widening access (WA) initiatives (otherwise known as pipeline, mentorship, outreach or academic enrichment programmes) which work with schools and communities that may not traditionally consider a career in medicine, to raise awareness about the career and support an application (Medical Schools Council 2014b). WA initiatives stem from governmental policies that aim to reduce discrepancies between the participation of different demographic groups of students in UK higher education (Connell-Smith and Hubble 2018). A wide variety of these programmes run across the UK (Greenhalgh et al. 2006; Kamali et al. 2005; Ratneswaran et al. 2015; Smith et al. 2013), the US (Crews et al. 2020; Martos et al. 2017; Navarre et al. 2017; Soto-Greene et al. 1999), Canada (Robinson et al. 2017; Rourke 2005) and Australia (Bennett et al. 2015; Gale and Parker 2013; Naylor et al. 2013).

School teachers are recognised as a key pathway through which pupils access information and initiatives about medical schools and medicine (Fleming and Grace 2014; McHarg et al. 2007; Medical Schools Council 2016) and their encouragement can positively influence pupils’ overall attainment and post-school choices (Alcott 2017; Gale et al. 2010). As such they are positioned as key stakeholders in the success of WA, acting as gatekeepers and influencers (Fleming and Grace 2014; Oliver and Kettley 2010). University students formally educated at UK state schools report, however, that poor quality guidance and lack of encouragement from teachers hindered their ability to make post-school choices (Mathers and Parry 2009; McHarg et al. 2007; Medical Schools Council 2013; UCAS 2015). These reports of poor teacher support parallel those in other countries with a focus on widening access to underrepresented groups, for example, the US, Canada and Australia (Behrendt et al. 2012; Fray et al. 2019; Gale and Parker 2013; Murray-García and García 2002; Restoule et al. 2013; Tomaszewski et al. 2017) however, studies on teachers’ influence on WA are limited. Medical students from state schools claim their teachers underestimated their chances of success (McHarg et al. 2007), gave them the impression they were destined for failure (Southgate et al. 2017), and that there was an anti-academic culture of low expectations in their schools (Mathers and Parry 2009). Other studies suggest that pupils, many of whom do have suitable attributes, may be put off medicine as they are not given the opportunity to imagine themselves as doctors, or lack the support to prepare themselves adequately for admission requirements (Greenhalgh et al. 2004; Southgate et al. 2015).

Medical schools are thus recommended to work more closely with teachers in disadvantaged schools (e.g., Medical Schools Council 2018) to prevent them becoming a barrier to WA initiatives and thus the diversification of the profession (Medical Schools Council 2014a). However, despite their role as influential stakeholders within WA, schoolteachers’ attitudes towards WA to medicine have been little explored in the literature. Although the aforementioned studies consider the influence of teachers on pupils’ aspirations and choices towards medicine from the point of view of pupils and medical students, only Southgate et al. (2015) included views from teachers (careers advisers) themselves. There is thus a substantial gap in understanding about exactly *how* medical schools should best engage and work with teachers, and why they may currently continue practices which discourage application to medicine. Without their support, potential applicants may leak out of the WA pipeline unnecessarily or feel encouraged to leave.

This study thus interviewed teachers advising pupils about medicine in high schools that were eligible for local medical schools’ WA initiatives to learn about their practices and attitudes, and to build insight into how and why they may be reluctant to promote medical school as an option to their pupils. Our overall aim was to build understanding and thereby consider how medical schools might better engage teachers as advocates for WA to medicine. However, the study also had a more critical purpose, in that we wished to consider why teachers themselves might find WA initiatives and their role within them problematic, and if so, how this impacted their practice. This was facilitated by the interpretation of results through the capability approach (Walker and Unterhalter 2010) which encourages educators to develop their students’ capability and thus freedom to choose their own future. The following research question guided the work: What do teachers in UK WA schools perceive to be their role in encouraging pupils to aspire to medicine and prepare an application?

## Methods

### Paradigm

This study is based within an interpretivist ontology, which acknowledged participants’ diverse and multiple realities, built relative to their social and cultural frames of reference and context (Crotty 2003). Epistemologically, the study follows social constructivism, which understands meaning to be co-constructed between researcher and participant, with the researcher striving to portray a credible and fair representation of participants’ experiences within this process of knowledge creation (Mann and MacLeod 2015). This paradigm allowed us to gather detailed and subjective accounts of participants’ perceived role and experiences, with a particular emphasis on their interpretations and context (Bunniss and Kelly 2010).

This approach understands the researchers to be a subjective and active element in all aspects of the research process, from conception to dissemination. Prior to obtaining a PhD in medical education, KA’s professional background was in designing and running university outreach and WA initiatives; JC is a psychologist; and SN is a clinical academic. All have experience and active roles in medical education research, with a particular interest in widening access to medicine and one author experienced a WA trajectory to higher education. To ensure that our perspectives did not dominate those of participants’ in the meanings constructed, we considered our positions and relationships with the data constantly and critically. For example, KA’s previous experience as a WA practitioner meant that she initially approached the study with assumptions built from this perspective: perceiving teachers as gatekeepers, either effective allies in boosting the uptake of initiatives if they positively encouraged pupils to participate, or problematic if they did not. However, during data collection and analysis, these assumptions were challenged and alternative roles for teachers were explored, discussed and justified. As a result, the paper considers a wider and more nuanced range of possible teacher roles, which go beyond this initial assumption.

### Data collection

#### Schools and participants

93.5% of school-aged pupils in the UK are educated at state-run schools (ISC 2020). High schools take pupils aged 11–18, whereas sixth-form colleges only educate pupils in their final years (16–18/19). The schools available to a child are strongly restricted by their location, and schools are thus often socioeconomically and socially segregated (Jerrim et al. 2017; The Challenge et al. 2017). These differences are reflected in school performance ratings (Scottish Government 2019; UK Government 2019). The current study aimed to capture a breadth of experiences from teachers working in a wide range of state-funded schools, which were also targeted by local medical schools for participation in WA initiatives. To be targeted for initiatives, schools had below average rates of progression to medicine and/or were situated in areas of socioeconomic deprivation.

Three medical schools (each in a different geographical region) approved the study and provided a list of the schools targeted by their WA initiatives. These lists were considered by the research team and a smaller purposive group of sixteen schools were targeted for an invitation to participate. This group was designed to contain the broadest possible diversity of schools: large to small; urban to rural; and with varying numbers of applicants applying to medicine each year (see Table 1). These decisions were informed using data publicly available on government websites.

**Table 1:** Participants’ professional role and school environment

School	Participant	Teacher’s primary role	School type	Location	Regional characteristics	Perceived aspiration of pupils to study*
A	1	Science Teacher	High School (ages 12–18)	Northeast Scotland	Rural; traditionally fishing, farming and factory-working communities; predominantly white communities	*At university*: Below average *Medicine*: Below average
B	2	Guidance Teacher				
B	3	Guidance Teacher				
B	4	Guidance Teacher				
C	5	Guidance Teacher				
D	6	Assistant Head of Sixth Form	Female only Sixth Form College (ages 16–19)	London	Urban; located in an area of high socioeconomic deprivation; large majority of pupils from minority ethnic communities	*At university:* Average *Medicine*: Above average
E	7	Engineering Teacher	Sixth Form College (ages 16–19)			
E	8	HE Progression Manager				
F	9	Careers Advisor	Sixth Form College (ages 16–19)	South England	Semi-urban; intake includes pupils from high and low socioeconomic areas; student body mixed ethnicities	*At university*: Average *Medicine:* Above average
F	10	Science Teacher				
G	11	Science Teacher				

*Although aspiration to study medicine may be above average, schools are targeted for WA initiatives according to their progression rates to university/medicine

Headteachers (principals) were sent the study information and an invitation for their school to participate. Of the sixteen schools contacted, eight headteachers gave permission for the study to be conducted in their school. Those who gave a reason for refusal, cited a lack of resources to allow teachers to participate in activities which did not directly benefit pupils. After headteachers had provided consent, teachers with responsibility for advising students on university choices (with a focus on medicine) were invited to participate by letter and email, and provided with a standardized information sheet via the headteacher or directly from the researcher. It is common in UK state schools for one teacher with a special interest to volunteer to be the key advisor for medicine, but their substantive roles may be very varied. Volunteers responded directly to the researchers by email to discuss the purpose and practicalities of the study, to be introduced to the researchers, ask any questions and, if happy to proceed, to arrange an interview. Thirteen teachers expressed interest and eleven wished to proceed to interview.

Our research design and objectives called for a small participant group, as we sought to understand and explore the range and complexity of these teachers’ perceptions—not to quantify or generalize them (Crouch and McKenzie 2006). Although a small-scale approach limits the transferability of findings, it preserves the individuality of teachers’ lived experiences, contexts and narratives, and allows any thematic similarities between them to be more fully explored.

#### Interviews

KA conducted semi-structured interviews at participants’ schools, following training on interviewing for research. KA introduced herself as a PhD student in medical education whose professional background was in the area of implementing WA initiatives, clarifying that she was not a doctor nor medical school staff to calibrate any perceived power imbalance and encourage participants to speak freely. A summary of the main questions for participants are included in Table 2. Participants were also encouraged to introduce topics they perceived to be of relevance which were not covered in the interview guide.

**Table 2:** Summary of key interview questions

Topic	Key questions
Background	
	What is your role at the school and how long have you worked here?
	Can you tell me about the school, the intake and surrounding area?
	Have you ever advised any pupils considering medicine? What was that like?
The pupils	
	You mentioned [pupils referred to previously]. Can you tell me more about them and their journey and decisions?
	How and when do you identify pupils for medicine? (age, process)
	Do you have pupils express an interest later on? Is there a difference in advising these pupils?
Teacher role/decisions	
	Have you ever had a student who you advised against applying or had reservations about? Why was this?
	How do you find reaching the right balance between supporting and more actively shaping decisions when advising the pupils?
	Thinking again of that balance, what role do other factors have as a responsibility for shaping decisions? (parents, peers, situation)
Outreach	
	When do pupils engage? How do you select pupils to participate?
	What are your experiences of outreach and its impact?

Participants were given the opportunity to ask questions about the study, were made aware that participation was entirely voluntary and that their consent could be withdrawn. All participants gave written consent to participate, for their interview to be audio recorded for transcription and for field notes to be made during interview.

#### Data analysis

Template analysis (King 2004) was used to organise and analyse data thematically and meaningfully show the relationships between themes as coding progressed. Template analysis acknowledges and incorporates the inevitable influence of prior literature and experience in the development of interpretations and pattern-identification, but remains largely data-driven, which was important within our exploratory study. The early introduction of structure, in the form of an initial template built on a subset of data, was beneficial however as it directed our focus to our specific research question. The template was refined and revised as coding progressed and remained flexible for unexpected insights to be included, thus maintaining an inductive character within the analysis.

Interview recordings were transcribed verbatim, anonymized, proofed for accuracy and imported to NVivo 11 (QSR International Pty Ltd, Doncaster, Vic, Australia) to organise and facilitate data analysis. KA undertook initial coding and discussed these codes with JC and SN at regular intervals throughout subsequent analytical cycles to ensure confirmability. Developing themes were scrutinised for trustworthiness and explored through critical conversation amongst the research team to explore, challenge and justify decisions taken.

#### Ethics

Permission to conduct the study was granted by the Committee for Research Ethics and Governance in Arts and Social Sciences and Business at the University of Aberdeen.

## Results

Eleven teachers participated in the study. They worked in seven schools across three regions of the UK (see Table 1). Collectively, teachers had an average of 10.5 years and a minimum of 4 years teaching experience. Interviews were one-to-one, except in one case, where two teachers were interviewed together at their request. Interview length averaged 28 min (range: 23–60 min).

The presentation of results explores how these teachers perceived their role in encouraging pupils to aspire to medicine and to prepare an application. It is structured in three sections, which explore themes related to:

1. Teachers’ attitudes towards their pupils’ aspiration to medicine.
2. Teachers’ perceived role in addressing contextual barriers during their pupils’ preparation for a medical school application.
3. Teachers’ perceived role in influencing pupils’ aspirations and decisions about application.

Later on, in the Discussion, these findings are situated within the wider literature on the experiences of pupils and critically analysed through the lens of the capability approach.

### Section 1: Attitudes towards pupils’ aspiration to medicine

Overall, teachers expressed a positive perception of medicine as a prestigious and worthwhile career. They talked warmly and proudly of former pupils who had entered medical school. Despite this generally positive view, however, teachers also communicated hesitations about their pupils aspiring to medicine, portraying this as a problematic or risky aspiration. Reasons for this were strongly context dependent but clustered around three main themes, explored below:

#### Aspiration due to family pressure

In schools where aspiration to medicine was moderate to high (see Table 1), teachers perceived that medicine was strongly prioritised by some families as an important marker of a child’s success. Teachers described numerous pupils who, at least initially, “choose medicine because they are told to” (Participant 7, London).

Although parental pressure was not seen as entirely detrimental (teachers reasoned it could also positively motivate pupils), all teachers strongly emphasised that they felt a genuine interest in the career of medicine was essential. They thus expressed deep concern when they felt a pupil’s aspiration to medicine was solely a reflection of their family’s wishes and were alert to watch out for this.

Teachers also perceived that a strong familial aspiration to medicine could prevent pupils from considering a range of other careers, including those that were more achievable or personally rewarding:

the two-fold challenge is that… not always does the aspiration meet real prospects, and that it can have the effect of shutting down other opportunities, other routes if students and their families are not careful. (Participant 8, London)

#### Unsuitable or uninformed motivations

Teachers reported that medicine was an obvious option for academically able pupils, and therefore if pupils chose not to pursue this career, it was not because they were unaware of it as a possibility. They felt that pupils were aware of the career from a young age and perceived some aspects to be appealing (e.g. the idea of helping people).

Teachers reported that, for some pupils the aspiration to study medicine was well motivated, however, that for others, these aspirations could be naïve or uniformed, and that the draw to medicine could be exacerbated by a pervasive hierarchy within healthcare subjects:

…talking to the students and it’s just, they kind of don’t want to fail and they see that as the thing, they see that as the pinnacle, medicine. (Participant 9, South England)

As result, teachers perceived that a minority of pupils aspired to medicine, not because the subject matter or career appealed, but rather to prove themselves as a high achieving student. Teachers saw this motivation as unsuitable, believing it to be untenable in terms of enjoying, excelling and persevering in a vocational career such as medicine.

#### Fear of not ‘making the grade’

Teachers were confident that many of their pupils had ample academic ability and suitable personal qualities to be successful in a medical course and career. However, they believed that even their high-fliers would struggle to achieve the academic entry requirements given the contextual factors in their schools and pupils’ home lives (see section “Methods”).

I do have students who come to me and I know they are never going to make the grade (Participant 10, South England)

Aspiration to medicine was thus implied to be unrealistic for many pupils because of the competitiveness of entry and the grade requirement, rather than because pupils were not capable of the career.

### Section 2: Preparation for medicine

This section explores how teachers helped pupils develop an aspiration for medicine and prepare for an application. Overall, teachers generally regarded the application process to medicine as daunting, long and emotionally difficult. The intense competition for places and the schools historic acceptance rates, meant that teachers framed the substantial time, energy and emotional commitment pupils required as a high-risk investment:

…but that’s something I can share with the other students, this is a long process: if you really want to do it, you are going to have to hang in there, it’s going to be a long process. (Participant 7, London)

#### Material constraints in schools

Teachers reported that it was their role to ensure pupils were aware of the academic and non-academic requirements for medicine. They acknowledged, however, that in addition to information, pupils required active support if they were to meet these entry requirements. Although many of the participating teachers voluntarily ran extra sessions to help support their pupils, they still expressed frustration and occasionally a sense of powerlessness about their ability to address the constraints imposed on pupils, for example: lack of support to prepare for the compulsory medical admissions tests; reduced teaching hours; limited subject choices; and staff shortages:

…the Chemistry teacher left, the Biology teacher left, the Physics teacher left, and [the pupils] ended up being taught by a deputy or the Head of Department who had practically no time… I know you’re supposed to do a lot of it yourself, but you do need some teacher input. Consequently, [our aspiring medic] didn’t apply for medicine, he pulled out at the last minute. (Participant 5, Northeast Scotland)

The schools often lacked staff, facilities and equipment, especially in science. Some schools resorted to restricting teaching hours to less than half those recommended for the course, or teaching pupils at different qualification levels together, to ensure provision. Rural schools banded together to collectively pool pupils and teaching staff to provide final year science in a central location. This approach was praised by one the teacher as it allowed high-achieving pupils to socialise with “like-minded others” and build their confidence (Participant 1, Northeast Scotland) especially as many pupils in the school were nervous about leaving home to attend university, an anxiety that precluded medical school for many rural pupils. However, as another pointed out, traveling several times a week to another town to attend class: “That’s tiring. You know, it’s not good” (Participant 5, Northeast Scotland).

All teachers directed pupils to sources of support where available, either in school and/or by local universities. In sixth-form colleges (large schools which take pupils aged 16-19 only—see Table 1), where there were greater numbers of potential applicants, teachers created and ran peer groups or preparation programmes for pupils aspiring to medicine and similarly classified subjects (e.g. Oxbridge, Law). One teacher described their programme as a tool to try to develop pupils’ skills and cultural awareness to the same level as happens naturally for more privileged pupils:

This is not something that you’re taught in a lesson… this is something that’s assimilated, and we’ve got to find mechanisms to kind of match what for most of these [more privileged] applicants will be almost a natural process of personal and academic development, including all those wider enrichments of culture and so forth that they often have access to. (Participant 8, London)

Acknowledging that their own time and school resources were limited, teachers also recognised these groups as an opportunity for pupils to provide each other with mutual support during the emotional and arduous selection process for medicine:

One of my biggest jobs is preparing them for possibly their first rejection in their lives, and really big rejection, and it could be serial rejections as well. And for high flying students… that is such a difficult thing to cope with… most of them haven’t done it before. And that really does knock back the confidence in all, well in all of them, it knocks them back. And so we have an awful lot of sharing and discussions and mutual support groups going on within [the programme], it’s a very, very open session, where people talk about the issues and the problems they are facing. (Participant 7, London)

Schools with small numbers of aspiring medics relied more heavily on universities’ outreach programmes to provide targeted preparation. Rural teachers were concerned pupils might be unable to afford the bus fare or be put off by the journey to the university, so one teacher described how she transported pupils to events in her own car. Participants’ accounts revealed the dedication of individual teachers to supporting aspiring medics, however, they also reported examples of schemes that had already been curtailed or discontinued. These practices thus appeared to be largely the preserve of committed individuals rather than institutionalised practices, and the prospect of a teacher’s retirement or the withdrawal of allocated time and resources to these programmes was a constant threat to their continuation.

Given these barriers, teachers considered exceptional drive, determination and a proactive attitude to be essential characteristics for medical aspirants.

#### Low confidence as a barrier

Teachers reported that their pupils often experienced difficulties and stressors related to living in low-income households (e.g. “overcrowding, parents perhaps, or grandparents, are unwell” (Participant 6, London)). Teachers thus found that their pupils demonstrated admirable resilience to achieve academically, keep calm and stay motivated “often despite incredible obstacles and challenges, not necessarily face by many of their peers in other areas” (Participant 8, London). They also reported, however, that many pupils experienced ‘imposter syndrome’ (Clance 1985) when imagining themselves as a potential medic and thus struggled to recognise and discuss their strengths, experiences and resilience as desirable traits for the career in their applications.

In schools where aspiration to medicine was not common, teachers reported that low confidence deterred pupils from openly committing to, and preparing for, a medical application. As a result, teachers were sometimes not aware that a pupil was interested, and thus did not know to offer them targeted information or support: “one girl we completely missed which was terrible” (Participant 5, Northeast Scotland).

One teacher reported pupils were “almost apologetic” for their interest in medicine, because they didn’t believe they would “be good enough” (Participant 4, Northeast Scotland). Another suggested revealing an aspiration for medicine in front of the class would invite ridicule from the peer group:

They’re not always confident enough to say: ‘I really want to do medicine, I’m determined to get these five As’ you know, because their mates will have a pop at them. Maybe just banter, maybe just jokes, but they’re not sturdy enough to handle that kind of banter, to have a response and say: ‘Yeah I am, what are you going to do?’ (Participant 3, Northeast Scotland).

Guidance teachers in these schools did have some protected time to discuss career choices with pupils one-to-one, however, this was typically limited to an annual ten-minute appointment. Teachers thus heavily relied upon informal conversations between pupils and subject teachers to provide careers aspiration and advice, rather than formal structures.

### Section 3: Influencing pupils’ decisions

This section discusses the strategies teachers employed to influence aspiration to medicine, and their approaches to advising pupils on whether or not to apply. In so doing, it explores our participants’ perception of the duties and boundaries of their role as an advisor.

#### Promoting opportunities to test motivations and suitability

Across all schools, teachers reported that their pupils usually volunteered an aspiration for medicine, rather than teachers suggesting this specifically to pupils. Moreover, in all but one school, teachers reported that when pupils revealed an aspiration to medicine, they acknowledged this as a positive career choice, but also simultaneously encouraged them to “think as creatively as possible” (Participant 11, South England) and *“*think of all you have and could possibly do” (Participant 9, South England). This strategy was intended to present other careers as equally valuable and interesting, and to combat familial pressure or the perceived hierarchy of medicine as the best or only career choice:

I’m heavily involved in trying to help promote sciences within the school and promote medical, biomedical sort of things to pupils. (Participant 1, Northeast Scotland)

Teachers considered it important to guide pupils to opportunities in which they could learn more about the realities of a medical career, to dispel naïve or inappropriate motivations and discover whether they would find the career enjoyable and rewarding. Some also felt a better understanding of medicine, and awareness of alternative careers, could help pupils build up enough confidence to resist parents’ wishes in favour of their own.

Teachers focussed their own support on helping pupils prepare their personal statement and interview skills: partly to produce a high-quality performance, but also further encourage pupils to explore and question whether they possessed the right skills and motivations. Familiarization with admissions tests (e.g. UKCAT, BMAT) were given the least attention.

#### Preserving a freedom to choose

For the reasons given above, teachers appeared cautious about exerting too much influence in a pupil’s decision about medicine. When advising about the career they thus tried to appear factual and if they did try to direct pupils’ aspirations, this was generally towards a group of subjects (e.g. the sciences or healthcare subjects) rather than medicine in particular:

I just tend to keep it all very factual: ‘this what you’ll need to do, this is the kind of skills they’re looking for’ and then they go away and make up their minds kind of thing. I don’t think I try and influence, I think I just try and inform. (Participant 4, Northeast Scotland)

Likewise, if a pupil decided not to pursue an interest in medicine, this was also usually accepted without a challenge:

Her Guidance teacher… he kind of said: ‘look, you should just give it a go and try these things’, but she came and she said, no, I don’t want to do it. So, that was that. So, you know, we can’t make them do it. (Participant 6, Northeast Scotland)

Although pupils decided against applying for medicine for a variety of reasons, most commonly this was because they had not achieved, or were not predicted to achieve, high enough grades. Teachers reported that pupils usually came to this decision by themselves, and with no need for teacher intervention.

When pupils were determined to apply without possessing the entry requirements, teachers felt, however, that it was their duty to ensure pupils knew their realistic chances: “so they can see that, you know, the odds are stacked against them” (Participant 11, South England). They found this part of their role challenging: “getting the realism at the right time when they can take it I suppose, I think, is the challenge sometimes” (Participant 9, South England).

Nonetheless, teachers strongly reported that “ultimately it has to be the student’s decision” (Participant 11, South England) and “I don’t dissuade them” (Participant 7, London). Rather, teachers saw their role as aiming to ensure pupils made their own decisions in an informed and realistic way. Teachers reported that this meant fewer, but stronger, candidates would apply, which was perceived positively.

## Discussion

Against a backdrop of increasing pressure on medical schools to widen access and diversify their cohorts (Mathers et al. 2011; McLachlan 2005; O’Neill et al. 2013) this study addresses a significant knowledge gap about *why* teachers in widening access (WA) schools have been reported as reluctant to promote medicine (Mathers and Parry 2009; McHarg et al. 2007; Medical Schools Council 2013; UCAS 2015).

We interviewed eleven teachers in a diverse range of UK high schools and sixth-form colleges targeted by WA initiatives, to better understand their perceived role in encouraging pupils to aspire to, and prepare, an application to medicine. Our findings show that teachers reported many of the same behaviours as pupils, and former pupils, have in previous studies. These included: warning pupils that it was going to be tough to apply to medicine (Mathers and Parry 2009); a belief that few pupils would be accepted (Southgate et al. 2017); and that teachers were unable to provide sufficient support because of material restraints within their schools (Mathers and Parry 2009; Robb et al. 2007; Southgate et al. 2015). Importantly, however, we found that our participants did not intend their actions to deter pupils from medicine. Instead, teachers reported that it was their role to guide pupils to improve their knowledge about the realities of medicine, as well as research alternative careers, and to build their capacity to make their own decisions about whether to apply: i.e. to inform, not influence.

### Teachers as advocates for widening access

Vitally, our study offers insight into *why* teachers held these perceptions. Despite the substantial time, energy and commitment some of our participants dedicated to supporting their pupils prepare for medicine, they readily acknowledged the limited capacity of their schools to compete with the advantages provided by more privileged institutions and families. Teachers described school environments in which the strongest, most resilient and dedicated applicants could succeed in their medical applications (and subsequently in their careers as doctors) whereas the majority would bow out pre-application.

It can thus be argued that teachers’ practices and attitudes are adapted to the structures they work within, and that an individualisation of blame onto teachers for setting low expectations detracts from the wider under-resourcing of schools and levels of deprivation experienced by certain neighbourhoods. As the Final Report of the Panel on Fair Access to the Professions states:

much careers advice is currently provided by staff who are full-time teachers, rather than professional advisers. It is not acceptable that the futures of young people rely on teachers having to provide advice and support above and beyond their normal teaching duties. While many teachers are well-meaning and dedicated to helping young people get on in life, careers advice is a professional and specialist service and should be operated on that basis (Panel on Fair Access to the Professions 2009)

We thus suggest that although there has been a cultural shift in terms of who can aspire to medicine [it is no longer seen as the sole preserve of the affluent and middle-class (Alexander et al. 2019)], there has not been an accompanying structural shift in secondary or tertiary education to allow less well-resourced aspirants the same opportunities to actually enter medicine as their more privileged peers.

Given these circumstances, we can also question whether teachers *should* be expected to be advocates for medicine. Encouraging stakeholders to challenge young people to aspire to careers not traditionally considered within their communities is at the heart of universities’ WA initiatives (Milburn 2012). The teachers in this study, however, rather erred towards a role in which they encouraged pupils to achieve and to broaden their horizons but did not wish to strongly direct or inspire them to any particular initiative or impart value-judgement on career choices. Teachers implied that their hands-off approach allowed pupils to be more informed and thus more capable of making an informed decision, but to still exert their own agency over decisions. This chimes with the capability approach, which argues a key goal of education should be to provide pupils with the capability and freedom to make rational decisions for themselves (Walker 2005). This theory is increasingly used to explore the relationship between social justice and education (Gale and Molla 2015; Walker and Unterhalter 2010; Wilson-Strydom 2015) and to evaluate WA interventions (Hart 2012; Walker 2008; Watts and Bridges 2006; Wilson-Strydom 2017). Moreover, it can be argued that it is morally and practically problematic for adults to ascribe particular visions of a ‘good’ future or career on younger generations, given the vast uncertainty and rapidly changing environment in which these generations will live as adults (Facer 2016).

There are, however, also legitimate criticisms of allowing pupils total freedom in their choice of career. For example, a more interventionist stance may be necessary to give pupils the confidence and opportunity to reveal their aspirations (Hart 2012), achieve the high requirements, and select a challenging and unfamiliar career such as medicine (Donnelly 2015; Oliver and Kettley 2010). A ‘hands off’ approach thus risks social stasis and allows disadvantage to be reproduced through the generations rather than challenged. Conceptualised within the capability approach, teachers’ accounts suggest that they saw WA initiatives primarily as additional resources for pupils to build their capability (i.e. skills, knowledge, support systems, aspiration, confidence) and thus be better positioned to realise their life and career goals; rather than to change the pupils’ aspirations per se. Teachers’ accounts also suggest they perceived it to be their role to be to direct pupils to these (and other) resources but not to push them to attend or engage. In other words, the choice whether to convert the resource to capability was largely left to the choice of the individual pupil. A problem thus arises, in that the capability to aspire is built out of resources, but if pupils are not encouraged to convert the resources available to them into a range of potential aspirations, they are instead likely to stick with those they already possess.

This study thus exposes a potential tension between the objectives of WA policy and the priorities of teachers. Although teachers were supportive of WA initiatives and medicine in general, they prioritised pupils’ freedom to choose whether to broaden their aspiration and opportunity set over the promotion of educational policies to increase social mobility.

### Strengths and limitations

We purposely interviewed teachers in a range of contexts, school types and teaching roles to include a wide breadth of perspectives. This allowed us to explore similarities and shared themes, as well as preserve the granularity of individual experiences. Although useful for our study purpose, the contextual differences and the small participant group add substantial limitations to the transferability of findings. We built measures into the research design to lessen teachers’ inhibitions about expressing critical views of medicine to a university researcher but acknowledge some participants may nevertheless have felt inhibited about speaking negatively about medicine given the study’s aims. Moreover, we can reasonably expect that less engaged teachers and those with a less positive view of medicine may have been less inclined to participate. Headteachers (principals) who declined to permission for their staff to participate cited under-resourcing as the major reason, which may mean schools in this situation were also less likely to be included.

Due to the restrictions of teachers’ busy schedules and/or the need to suddenly attend to pupils, some interviews were cut short, with implications for the quantity of data. Two teachers were interviewed together, which may have inhibited their willingness to give a full account of their experiences, fearing the judgement of the other. On the other hand, being interviewed together was their choice, which might have made the situation more comfortable and hence generated deeper and more nuanced contributions from both (Finch et al. 2014).

There may be a greater tendency within a small participant group for some voices to disproportionately influence interpretations. To address this possibility, we made a conscious effort to give equal weight to each participant’s account in analysis and write-up, despite their different levels of experience and eloquence. The inclusion of participant validation may have further boosted the credibility of the work, however, we decided against this, primarily to not add more to teachers’ workload and because we thought the commitment to repeated contact might be a barrier to recruitment.

Practicing reflexivity, as described in the methods, as well as critical discussions amongst the research team were used to minimize potential researcher bias. The diverse professional backgrounds within the team was a strength in this regard, as members approached the data and topic from differing perspectives. There were limits to this diversity however, as all researchers now identity as female, white and middle-class, which is typical for medical education researchers in this area.

### Implications for research and practice

Comparison studies to investigate the perceptions and strategies of teachers from a variety of schools (including grammar and independent, as well as less-engaged WA eligible state schools) would offer interesting insight into teachers’ approaches and perception of role in different contexts. Furthermore, given the parallels between the UK and other countries, where teachers work in similarly under-resourced environments and promote similarly conservative messages about medicine (Southgate et al. 2015), the study aims and findings may warrant further investigation in other contexts. Comparison studies that include both teacher and their pupils’ voices would also be beneficial: in this study we are limited to the exploration of teachers’ accounts and cannot comment on pupils’ interpretation of their behaviour.

As described above, teachers appeared to conceptualise their role as facilitating pupils’ access to WA to medicine resources, rather than as a resource themselves. This concurs with the Access to the Professions report (quoted above), which essentially argues it is not appropriate to require a science teacher to also be an expert advisor on medicine. Our study reveals how these lines are often blurred however: pressure to compensate for lacking resources results in dedicated teachers taking it upon themselves to create preparation programmes and provide pastoral support. As a result, we suggest open discussions between WA stakeholders to agree a clear alignment and delimitation of stakeholder roles. If agreed, medical schools might then focus on supporting teachers to facilitate access to expert resources through improved communication and information sharing networks; whilst medical schools provide this expert resource.

Medical schools may also wish to support teachers to be more proactive in encouraging a wider range of pupils to engage with the resources that are available, to enable the possibility of new aspirations, as well as opportunities, to be built. We suggest that medical schools and policy makers thus consider ways in which they can realign systems to make promoting medicine appear a less risky choice to teachers. Medical schools could consider how to engage teachers in a dialogue about how they can best support pupils to highlight their strengths (e.g. intellectual ability, commitment, resilience) in an application, as well as inform and reassure teachers about some of the realities of medical application (e.g. around acceptance rates and re-application) in a process of myth-busting. This could be combined with heightening teachers’ awareness of alternative paths to medicine, including foundation programmes and gateway courses. More fundamentally, medical schools might also consider working with teacher professional development and training teams, to receive expert advice on how they might help teachers recognise the constraints of a well-intentioned belief in prioritising a pupil’s individual choices.

## Conclusions

Overall, this study reaffirms the contextual barriers present daily in WA schools, and reveals original insight into how these may influence teachers’ perceptions of their role. It also highlights these teachers’ prioritisation of pupils making their own choice of career. We suggest that the expectation for teachers in WA schools to advocate medicine cannot happen in isolation and instead that this requires the support of other stakeholders, policies and systems aligning to provide their pupils with opportunities to develop their capability, as well as better chances of actually achieving a place (Alexander and Cleland 2018; Gorman 2018). Acknowledging and understanding teachers’ concerns and working with them to construct better partnerships and a more positive view of the journey to medicine, might be a tangible start (Greany et al. 2014).

## References

[CR1] AFMC. (2010). *The future of medicine in Canada*. The Association of Faculties of Medicine of Canada; Ottawa. https://www.afmc.ca/future-of-medical-education-in-canada/medical-doctor-project/. Retrieved 20 October 2015.

[CR2] Alcott B (2017). Does teacher encouragement influence students’ educational progress? A propensity-score matching analysis. Research in Higher Education.

[CR3] Alexander K, Cleland J, Shah M, McKay J (2018). Social inclusion or social engineering? The politics and reality of widening access to medicine in the UK. Achieving equity and quality in higher education.

[CR4] Alexander K, Cleland J, Nicholson S (2019). Bridging the cultural divide? Exploring UK school pupils’ perceptions of medicine. Medical Education.

[CR5] Behrendt, L., Larkin, S., Griew, R., & Kelly, P. (2012). *Review of higher education access and outcomes for aboriginal and torres strait Islander People.* https://docs.education.gov.au/system/files/doc/other/heaccessandoutcomesforaboriginalandtorresstraitislanderfinalreport.pdf. Retrieved 5 March 2016.

[CR6] Bennett, A., Naylor, R., Mellor, K., Brett, M., Gore, J., Harvey, A., et al. (2015). *Critical interventions framework part 2. Equity initiatives in Australian higher education: A review of evidence of impact.* Canberra. https://nova.newcastle.edu.au/vital/access/manager/Repository/uon:32946

[CR7] Bolton P (2017). Grammar school statistics: Number 1398. London.

[CR8] Bunniss S, Kelly DR (2010). Research paradigms in medical education research. Medical Education.

[CR9] Clance PR (1985). The impostor phenomenon.

[CR10] Connell-Smith, A., Hubble, S. (2018). Widening participation strategy in higher education in England. The House of Commons Library; London. https://commonslibrary.parliament.uk/research-briefings/cbp-8204/. Retrieved 29 May 2020.

[CR11] Crews DC, Wilson KL, Sohn J, Kabacoff CM, Poynton SL, Murphy LR (2020). Helping scholars overcome socioeconomic barriers to medical and biomedical careers: Creating a pipeline initiative. Teaching and Learning in Medicine.

[CR12] Crotty M (2003). The foundations of social research: meaning and perspective in the research process.

[CR13] Crouch M, McKenzie H (2006). The logic of small samples in interview-based qualitative research. Social Science Information.

[CR14] Department of Education. (2019). *Undergraduate applications, offers and acceptances, 2018.* Australian Government; Canberra, AC. https://docs.education.gov.au/node/51541. Retrieved 13 July 2019.

[CR15] Donnelly M (2015). A new approach to researching school effects on higher education participation. British Journal of Sociology of Education.

[CR16] Facer K, Lees H, Noddings N (2016). Using the future in education: Creating space for openness, hope and novelty. The Palgrave international handbook of alternative education.

[CR17] Finch H, Lewis J, Turley C, Ritchie J, Lewis J, Nicholls CMN, Ormston R (2014). Focus groups. Qualitative research practice: A guide for social science students and researchers.

[CR18] Fleming MJ, Grace DM (2014). Widening the lens: Utilizing teacher perspectives to assess widening participation efforts in Australian higher education. Widening Participation and Lifelong Learning.

[CR19] Fray, L., Gore, J., Harris, J., & North, B. (2019). Key influences on aspirations for higher education of Australian school students in regional and remote locations: a scoping review of empirical research, 1991–2016. *The Australian Educational Researcher*, Early Online; pp. 1–33. 10.1007/s13384-019-00332-4.

[CR20] Gale T, Molla T (2015). Social justice intents in policy: An analysis of capability for and through education. Journal of Education Policy.

[CR21] Gale, T., & Parker, S. (2013). *Widening participation in australian higher education: Report submitted to HEFCE and OFFA.* http://www.ncsehe.edu.au/wp-content/uploads/2013/10/2013_WPeffectivenessAus.pdf. Retrieved 4 September 2017

[CR22] Gale, T., Sellar, S., Hattam, R., Comber, B., Tranter, D., & Bills, D. (2010). *Interventions early in school as a means to improve higher education outcomes for disadvantaged students*. Australian Government: Canberra, AC. http://dro.deakin.edu.au/view/DU:30040776. Retrieved 11 June 2018

[CR23] Gorman D (2018). Matching the production of doctors with national needs. Medical Education.

[CR24] Greany, T., Gu, Q., Handscomb, G., Varley, M., Manners, P., & Duncan, S. (2014). *School*-*university partnerships: Fulfilling the potential.* https://www.publicengagement.ac.uk/sites/default/files/publication/supi_project_report_final.pdf. Retrieved 8 September 2019.

[CR74] Greenhalgh T, Russell J, Boynton P, Lefford F, Chopra N, Dunkley L (2006). “We were treated like adults” - development of a pre-medicine summer school for 16 year olds from deprived socioeconomic backgrounds: action research study. BMJ.

[CR25] Greenhalgh T, Seyan K, Boynton P (2004). “Not a university type”: Focus group study of social class, ethnic, and sex differences in school pupils’ perceptions about medical school. BMJ.

[CR26] Hart CS (2012). Aspirations, Education and Social Justice: Applying Sen and Bourdieu.

[CR27] ISC. (2020). Annual census. Independent schools council; London. https://www.isc.co.uk/research/annual-census/ Retrieved 24 Apr 2020.

[CR28] Jerrim, J., Shure, N., Gutiérrez, G., Torres, R., Shure, N., Gutiérrez, G., & Torres, R. (2017). Socioeconomic segregation in secondary schools. *In The PISA effect on global educational governance* (pp. 95–108). London: Routledge. 10.4324/9781315440521-7.

[CR29] Kamali AW, Nicholson S, Wood DF (2005). A model for widening access into medicine and dentistry: The SAMDA-BL project. Medical Education.

[CR30] King N, Cassell C, Symon G (2004). Using templates in the thematic analysis of text. Essential guide to qualitative methods in organizational research.

[CR31] Larkins S, Michielsen K, Iputo J, Elsanousi S, Mammen M, Graves L (2015). Impact of selection strategies on representation of underserved populations and intention to practise: international findings. Medical Education.

[CR32] Mann K, MacLeod A, Cleland J, Durning SJ (2015). Constructivism: Learning theories and approaches to research. Researching medical education.

[CR33] Martos AJ, Piracha YS, Oladele M, Erves JG, Dorn J, Friedman E (2017). An innovative educational pipeline programme for under-represented youth: The Sophie Davis Biomedical Education/CUNY School of Medicine model. Education for Primary Care.

[CR34] Mathers J, Parry J (2009). Why are there so few working-class applicants to medical schools? Learning from the success stories. Medical Education.

[CR35] Mathers J, Sitch A, Marsh JL, Parry J (2011). Widening access to medical education for under-represented socioeconomic groups: Population based cross sectional analysis of UK data, 2002–2006. BMJ.

[CR36] Mathers J, Sitch A, Parry J (2016). Population-based longitudinal analyses of offer likelihood in UK medical schools: 1996–2012. Medical Education.

[CR37] McHarg J, Mattick K, Knight LV (2007). Why people apply to medical school: Implications for widening participation activities. Medical Education.

[CR38] McLachlan JC (2005). Outreach is better than selection for increasing diversity. Medical Education.

[CR39] Medical Schools Council. (2013)*. Selecting for excellence: End of year report*. http://www.medschools.ac.uk/Publications/Documents/MSC-Selecting-for-Excellence-End-of-year-report.pdf. Retrieved 15 July 2015.

[CR40] Medical Schools Council. (2014a). *Selecting for Excellence: Final Report*. http://www.medschools.ac.uk/SiteCollectionDocuments/Selecting-for-Excellence-Final-Report.pdf Retrieved 15 July 2015.

[CR41] Medical Schools Council. (2014b)*. A journey to medicine: Outreach guidance*. https://www.medschools.ac.uk/media/1913/a-journey-to-medicine-outreach-guidance.pdf. Retrieved 15 July 2015.

[CR42] Medical Schools Council. (2016). *Implementing selecting for excellence a progress update*. http://www.medschools.ac.uk/SiteCollectionDocuments/Selecting-for-Excellence-2016-update-MSC.pdf. Retrieved 1 August 2016.

[CR43] Medical Schools Council. (2018). *Selection alliance 2017 report: An update on the medical schools council’s work in selection and widening participation.* https://www.medschools.ac.uk/media/2536/selection-alliance-2018-report.pdf. Retrieved 12 January 2019.

[CR44] Milburn, A. (2012). *Fair access to professional careers.* The Independent reviewer on Social Mobility and Child Poverty; London. https://www.gov.uk/government/uploads/system/uploads/attachment_data/file/61090/IR_FairAccess_acc2.pdf. Retrieved 2 December 2015

[CR45] Murray-García JL, García JA (2002). From enrichment to equity: Comments on diversifying the K-12 medical school pipeline. Journal of the National Medical Association.

[CR46] Navarre B, Perez NA, Smith ST (2017). Constructing an online MCAT preparation program as an alternative to on-site preparation for medical school. Journal of Education and Learning.

[CR47] Naylor R, Baik C, James R (2013). A critical interventions framework for advancing equity in Australian higher education.

[CR48] O’Connell TF, Ham SA, Hart TG, Curlin FA, Yoon JD (2017). A National Longitudinal Survey of Medical Students’ intentions to practice among the underserved. Academic Medicine.

[CR49] O’Neill L, Vonsild MC, Wallstedt B, Dornan T (2013). Admission criteria and diversity in medical school. Medical Education.

[CR50] Oliver C, Kettley N (2010). Gatekeepers or facilitators: the influence of teacher habitus on students’ applications to elite universities. British Journal of Sociology of Education.

[CR51] Panel on Fair Access to the Professions. (2009). *Unleashing aspiration: The final report of the panel on fair access to the professions.* London. http://dera.ioe.ac.uk/5284/1/finalreport.pdf. Retrieved 26 July 2017.

[CR52] Puddey IB, Playford DE, Mercer A (2017). Impact of medical student origins on the likelihood of ultimately practicing in areas of low vs. high socio-economic status. BMC Medical Education.

[CR53] Ratneswaran C, Mushtaq J, Steier J (2015). A model for medical school application courses: Widening access to student preparation. Medical Education.

[CR54] Restoule, J.-P., Mashford-Pringle, A., Chacaby, M., Smillie, C., Brunette, C., & Russel, G. (2013). Supporting successful transitions to post-secondary education for indigenous students: Lessons from an institutional ethnography in Ontarion, Canada. *The International Indigenous Policy Journal*, 4(4). https://core.ac.uk/download/pdf/26922846.pdf. Retrieved 8 September 2019.

[CR55] Robb N, Dunkley L, Boynton P, Greenhalgh T (2007). Looking for a better future: Identity construction in socio-economically deprived 16-year olds considering a career in medicine. Social Science and Medicine.

[CR56] Robinson MA, Douglas-Vail MB, Bryce JN, Van Zyl TJ (2017). Medical school outreach and mentorship for rural secondary school students: A pilot of the Southwestern Ontario Medical Mentorship Program. Canadian Journal of Rural Medicine.

[CR57] Rourke J (2005). Strategies to increase the enrolment of students of rural origin in medical school: Recommendations from the Society of Rural Physicians of Canada. CMAJ.

[CR58] Scottish Government. (2019). Achievement of curriculum for excellence levels: 2018–2019. https://www.gov.scot/publications/achievement-curriculum-excellence-cfe-levels-2018-19/. Accessed 1 May 2020.

[CR59] Smith S, Alexander A, Dubb S, Murphy K, Laycock J (2013). Opening doors and minds: A path for widening access. Clinical Teacher.

[CR60] Soto-Greene M, Wright L, Gona OD, Feldman LA (1999). Minority enrichment programs at the New Jersey Medical School. Academic Medicine.

[CR61] Southgate E, Brosnan C, Lempp H, Kelly B, Wright S, Outram S, Bennett A (2017). Travels in extreme social mobility: How first-in-family students find their way into and through medical education. Critical Studies in Education.

[CR62] Southgate E, Kelly BJ, Symonds IM (2015). Disadvantage and the “capacity to aspire” to medical school. Medical Education.

[CR63] Steven K, Dowell J, Jackson C, Guthrie B (2016). Fair access to medicine? Retrospective analysis of UK medical schools application data 2009–2012 using three measures of socioeconomic status. BMC Medical Education.

[CR64] The Challenge, iCoCo, & SchoolDash. (2017). *Understanding School Segregation in England; 2011 to 2016*. http://the-challenge.org/uploads/documents/TCN-Understanding-School-Segregation-in-England-2011-to-2016.pdf. Accessed 24 March 2017.

[CR65] Tomaszewski W, Perales F, Xiang N (2017). School experiences, career guidance, and the university participation of young people from three equity groups in Australia.

[CR66] UCAS. (2015). *Through the lens of students: How perceptions of higher education influence applicants choices.* https://www.ucas.com/sites/default/files/through-the-lens-of-students.pdf. Retrieved 14 July 2017.

[CR67] UK Government. (2019). All schools and colleges in England—Find and compare schools in England. https://www.compare-school-performance.service.gov.uk/schools-by-type?step=phase&geographic=all&region=0&phase=secondary. Accessed 5 May 2020

[CR68] Walker, M. (2005). Amartya Sen’s capability approach and education. *Educational Action Research (Vol. 13)*. https://www.tandfonline.com/doi/pdf/10.1080/09650790500200279. Retrieved 27 July 2018.

[CR69] Walker M (2008). Widening participation; widening capability. London Review of Education.

[CR70] Walker M, Unterhalter E (2010). Amartya Sen’s capability approach and social justice in education.

[CR71] Watts M, Bridges D, Deneulin S, Sagovsky N (2006). Enhancing students’ capabilities? UK Higher Education and the widening participation Agenda. Transforming unjust structures: The capability approach.

[CR72] Wilson-Strydom M (2015). University access and theories of social justice: Contributions of the capabilities approach. Higher Education.

[CR73] Wilson-Strydom M, Mountford-Zimdars A, Harrison N (2017). Widening access with success: Using the capabilities approach to confront injustices. Access to higher education: Theoretical perspectives and contemporary challenges.

